# The Contribution of the Humanities and Social Sciences to Pharmacy Education: Literature Review and Perspectives

**DOI:** 10.3390/pharmacy8040227

**Published:** 2020-11-24

**Authors:** Caroline Boulliat, Emily Darlington, Marie-Ange Faure, Bernard Massoubre, Claude Dussart

**Affiliations:** 1 Pharmacie à Usage Intérieur and Hôpital d’Instruction des Armées Desgenettes, 69003 Lyon, France; marie-ange.faure@intradef.gouv.fr (M.-A.F.); bernard.massoubre@intradef.gouv.fr (B.M.); 2 Laboratoire Parcours Santé Systémique (P2S) EA 4129 and Université Claude Bernard Lyon 1, 69372 Lyon, France; emily.darlington@univ-lyon1.fr; 3 Pharmacie et Stérilisation Centrales and Hospices Civils de Lyon, 69230 Sainte-Genis-Laval, France; claude.dussart@univ-lyon1.fr

**Keywords:** social sciences, pharmacy, education, professional training

## Abstract

Background: Healthcare systems worldwide adapt to patients’ needs and expectations, following social evolutions. Pharmaceutical practice has shifted towards activities such as therapeutic education. Such new missions require to prioritize human and social sciences, which now play a predominant role in training. Objective: This work consists of assessing the contribution of human and social sciences to the field of pharmacy, with a twofold focus on practice and training. Method: A literature review was carried out according to the PRISMA guidelines focusing on the last 10 years. Selected full texts were read and analyzed to elicit the contribution of human and social sciences to pharmacy. Results: Overall 36 articles were included. Three specific topics were identified during an inductive process of full text analysis: public health policy, patient care, and interprofessional collaboration. Conclusions: Although human and social sciences are essential to the evolution of the pharmacist profession, their impact on health care costs remains difficult to evaluate. Moreover, teaching human and social sciences can prove difficult to standardize. Such approaches must be supported and organized by governments and universities with a view of upscaling practices.

## 1. Introduction

With a view to promote and foster the health of populations, healthcare systems evolve in line with the needs of patients and the societal demands related to care and population health. To this end, many policy reforms and changes are being prescribed and implemented worldwide, among which the promotion of intersectoral practices. One of the key strategies has been to incorporate public health into pharmaceutical practices. As S. Dindial et al. wrote in 2012, pharmacists as community health professional play an important role in public health, as they can educate patients and optimize the use of medications [1]. The health of population is indeed dependent on the competencies of community public health professionals [2]. Furthermore, as pointed out by WA Zellmer in an interview of Golodner in 2013 about pharmacy skills and pharmacy education [3], interprofessional work is a cornerstone of public health strategies. This author also highlights the value of including good communication as a target for training in pharmacy education. This will be particularly beneficial to foster the very much needed links between community and hospital practices. The evolution of the pharmacist profession, has oriented missions towards therapeutic education and interprofessional work. Activities such as drug reconciliation implies that pharmacists should demonstrate different skills, which are not limited to technical, scientific knowledge required by basic scientific practice. Thus, human and social sciences are essential in pharmacy. Our work has consisted in assessing the extent to which this field is likely to be integrated into pharmacy practices and consequently into the initial training of practitioners. In this respect, in 2018, TI Poirier et al. called for innovation “in pharmacy education to enhance compassion and caring, through an emphasis on the value of social sciences and humanities, as our students develop into health care practitioners”. [4] TI Poirier et al. share the need and desire to upscale the pharmacist profession. We argue that it is thus essential to characterize what contribution human and social sciences (HSS) can make to pharmaceutical practices, particularly during professional training.

## 2. Materials and Methods

A review of the literature was conducted to assess the contribution of human and social sciences to pharmacy training and the perspectives relating to the contribution of human and social sciences to pharmacy practices.

The MEDLINE database was searched using the following keywords: “social sciences”, “humanities” and “education, pharmacy”.

The MEDLINE equation was

((((social [All Fields] AND (“pharmacy” [MeSH Terms] OR “pharmacy” [All Fields] OR “pharmacies” [MeSH Terms] OR “pharmacies” [All Fields]) OR “social sciences” [MeSH Major Topic])) OR “humanities” [MeSH Major Topic]) AND “education, pharmacy” [MeSH Major Topic] AND (“2009/01/01” [PDAT] : “2019/05/01” [PDAT])

An initial search was conducted to identify existing articles and two articles from this search were used as positive controls to validate our MEDLINE search equation.

The search was limited to articles published between January 2009 and 1 May 2019 in order to obtain current and timely articles which would be informative in constantly evolving policy contexts as mentioned above. The search was not limited to in terms of language of publication, as both French and English papers were included.

A second round of searching was carried out through the screening of the bibliography from selected articles which had been identified as responding to our search criteria. Lists of references were analyzed to identify relevant articles which had not been identified in the initial search.

The titles and abstracts from selected articles were methodically analyzed against inclusion/exclusion criteria and a list was made and exported to an Excel file for further analysis. Articles which met all inclusion criteria were then selected for full text analysis. Articles were excluded from a full text analysis if the abstract met at least one of the following criteria: humanities and social sciences were addressed as a tool for teaching, the focus of the article concerned the internal organization of education (evaluation, mentoring, funding, and faculty), or access to pharmacy education, international exchanges during studies, continuing education, and use of social networks as a course support.

Our analysis consisted in a traditional/narrative literature review. At each stage of article selection, the results of each author’s analysis were collated and shared in a multi-stakeholder consultation to analyze differences and reach consensus. Articles written in Japanese were excluded from our research; their translation seemed difficult to us and exposed us to misinterpretation. In addition, we chose to focus on the more abundant English literature in this field; so a potential bias exists in our approach.

The group of authors met after the reading of the included complete articles in order to validate the common themes identified by the 2 readers: each article was thus taken up again to check the coherence of the identified themes and their distribution. In case of conflict, the article was placed by default in the 3 themes; nevertheless this did not happen: an agreement was systematically reached between the authors.

The work was then distributed among the authors according to this organization, this time for a thematic analysis of the articles. This enabled the authors to compare the different approaches, which promoted a more meaningful and relevant discussion on a limited number of articles [5].

## 3. Results

A total of 364 articles were selected in the first round of screening. From these, 175 abstracts were selected for further review, and 189 articles were excluded. A total of 36 references were selected for full text analysis and are included in the results below. Out of these, 11 full-text articles were excluded because they were written in Japanese.

**Figure 1:**
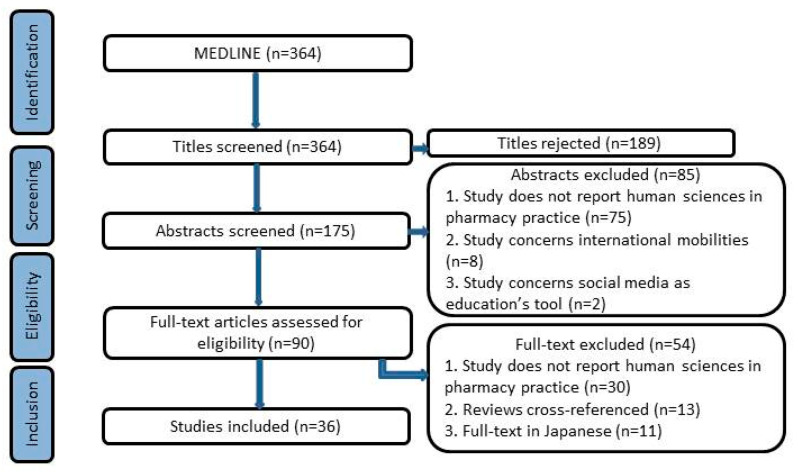
Flow diagram of study inclusion and exclusion.

A flowchart summarizing the selection process for the study is reported in Figure 1.

Appendix A summarizes the characteristics of the article: year of publication, type of publication, country, and main topic addressed. 22 texts are empirical studies of which 72.7% (n = 16) are American.

We found that 44.4% (n = 16) of the articles were concerned with knowledge of how to relate to the patient, 22.2% (n = 8) focused on interprofessional collaborations and the integration of a multidisciplinary team, and 33.3% (n = 12) on the strategic evolution of the pharmacist profession.

From analysis of complete texts, the main themes addressed were extracted: public health policy (including public health and the evolution of professional practice for patient-centered care), relationship skills towards the patient (including professionalism, ethics and cultural competences), and finally interprofessional collaboration and integration of a multidisciplinary team [6].

In depth analysis of each theme in focus as presented below.

### 3.1. Strategic Evolution of the Pharmacist Profession

Out of 12 articles addressing this topic, 25% (n = 3) are comparative studies, and all were American. The articles are dated from 2009 to 2017 which highlights that the evolution of the profession is not new and has been adapting to legislative and regulatory requirements, and that it is still relevant today; it also evolves in relation to the health of populations, taking into account the health crises it may encounter. Human and social sciences therefore provide pharmacists with the new skills they need to adapt to such new requirements, which are focused on comprehensive patient care. The latter is now intended to be multidisciplinary: pharmacists must therefore learn to work in collaboration with other health professionals (and with the patient as well), thus putting into practice communication skills.

#### 3.1.1. Evolution of the Pharmacist Profession and Initial Training of Practitioners

In 2009, Momas [7] recalls the traditional definition of public health: it is a discipline of action aimed at protecting, promoting, and restoring the health of populations. Pharmacists are presented as public health stakeholders who must be trained at a graduate and post-graduate level. Indeed, as pharmacists are at the heart of multidisciplinary public health teams, they must be trained to communicate with patients, to collaborate with other health professionals, public authorities and social workers. In addition, pharmacists have a privileged role in informing, advising and educating patients, as well as a role in pharmacovigilance and drug iatrogeny. However, the pharmacist’s role differs according to their place of practice (pharmacy, biology laboratory, or hospital). Moreover, the author highlights the critical situation of public health training: although its teaching is a necessity, it is difficult to identify trained teachers.

In 2010, David and Locher [8] revisit this evolution of the pharmacist profession in France, in relation to the policy and legislative framework. The authors highlight the relevance of human and social sciences as a contribution to adapt pharmacist practices on at least three levels: understanding patients and their living conditions (e.g., social and geographical environment), the interaction and coordination of a dynamic interprofessional network, the different modes of regulation of medicines at the political, and economic and social levels. In this context, the reform of the French pharmacy faculties is partly inspired by the American reform. Pharmacists must take their place in their professional network, which they must know of, and understand, the determinants of health. The authors conclude that the integration of human and social sciences throughout the pharmacist’s training could make it possible for the students to acquire the skills required by the new regulations: skills needed vis-à-vis the patient and his or her therapeutic education; skills needed vis-à-vis other health professionals. This could also be beneficial for the independence and autonomy of the profession.

As early as 2011, Offiong et al. [9] show us that American university programs evolve in line with the Healthy People 2020 recommendations by developing city/hospital relationships and adapting training to be more person-centered and prevention.

#### 3.1.2. Evolution of the Profession and Global Disparities

In this area, the developments vary from one country to another: they generally follow the same dynamics, but it should be noted that there are certain global disparities, which affect the status of the profession itself and the initial training of de facto practitioners that is carried out.

In 2011, two articles describe the differences between developing and developed countries in the use of humanities and social sciences in pharmacy practices [10,11]. Indeed, Hassali et al. point out that the conceptions of the pharmacist profession has not changed in developing countries and that pharmacists are still perceived only as drug sellers. While the need for human and social sciences in patient care is well established in developed countries [10], their importance is only beginning to emerge in developing countries [11]. Hassali et al. also point out that the high level of conflict between pharmacists and other health professionals in developing countries has had an impact on the evolution of the pharmacist profession.

In 2017, Law et al. [12] review the importance of pharmacists in public health despite global disparities. Indeed, although prevention occupies a major place in developed countries, it must be reinforced in developing countries, especially primary prevention. The experience in developed countries, from a hundred years in the USA and Canada, shows, however, the lasting effect of public health, particularly on health costs. The difference between developed and developing countries has been observed for a long time. Indeed, Youmans et al. [13] highlight a number of problems as early as 2012 in Tanzania. Although clinical pharmacy students are part of a multidisciplinary team, there is a need to make a shift in the curriculum so that students receive training which is adapted and in line with the local needs. Indeed, pharmacists currently provide drugs but could also be involved in vaccination, in the prevention of infections. This article thus highlights a marked difference with the United States where the profession is more patient-centered than in Tanzania.

Disparities in the implementation of such prevention policy in developed countries also exist. This is what Nunes-da-Cunha et al. [14] show in 2016. By comparing the number of hours of patient-centered classes between the United States and Europe, they show that there are more patient-centered classes in the United States than in Europe and that disparities within Europe also exist. They therefore conclude that in order to follow the recommendations directed towards policy makers, European countries must continue to review their curricula. The United States is ahead of the rest of the developed world in this matter: the 2013 article from the American College of Clinical Pharmacy [15] presents that this is related to the increased diversity in social and nationality backgrounds in the United States. Nurses pioneered patient-centered care with discussions on cross-cultural nursing in the 1980s; physicians then included it in their training program in the late 1990s. For pharmacists, this appeared in the discussions in the 1990s, but it was at least 10 years before this was introduced into teaching. The United States are already beginning to evaluate the quality of the courses provided: courses still need to be standardized between the different faculties to ensure a certain level of homogeneity in the knowledge provided between practitioners. Moreover, the content of these courses should be evaluated to identify their impact on healthcare costs. The importance of evaluation is also highlighted by King et al. [16] in their 2015 study. The latter shows the importance of introducing students from other professional fields to these courses as professionalism is addressed from the very beginning of the course, which was highlighted by Teagarden in 2013 [17].

With the aim of improving programs and of developing the pharmacist profession in accordance with the international recommendations in line with the evolution of patients’ health-related needs, Bush et al. [18] address student organizations in their paper. The authors present the importance of knowing the values of such organizations in order to adapt programs and curricula, and involve students in the evolution of their profession.

### 3.2. Relationship Skills Towards the Patient

There are 16 articles which deal with the necessary relationship skills with and towards the patient. The following topics are addressed: cultural competence (n = 8), professionalism (n = 2), cultural competence and professionalism (n = 3), ethics (n = 1), and ethics and professionalism (n = 2). Twelve of these 16 articles are studies of which 83% (n = 10) are American. The articles identified in this theme deal with the skills required by pharmacists to take into account patients’ origins or beliefs in patient care. Such elements even are likely to influence practitioners’ practices and therefore their relationship with the patient. Insofar as the profession is oriented towards therapeutic education activities, cultural and emotional skills must be taken into account and integrated into professional training. The articles we identified set out some of the aspects including in training and how they are taught particularly in the United States. It can be noted that the United States are ahead of the game in integrating human and social sciences into their students’ training programs. The notion of professionalism is also developed here; the pharmacist’s attitudes must be adapted to patients, pharmacists must take into account patients’ pathology and knowledge. Furthermore, health ethics has recently been integrated into pharmaceutical studies; its teaching must be reinforced. It must encompass the evolution of technologies and the new means of communication used by health professionals.

#### 3.2.1. Cultural and Emotional Competencies

In 2017, an American report by Godwin Jr. et al. [19] recalls the definition of cultural competence as, “the ability of individuals and systems to respond respectfully and effectively to people of all races, ethnicities, genders, gender identities, sexual orientations, ages, social classes, physical abilities or attributes, religious or ethical value systems, national origins, and political beliefs in a way that recognizes, affirms and values cultural differences and similarities and the worth of individuals, families, and communities and protects and preserves the dignity of each one of them.” The authors reiterate that such competencies are an integral part of a pharmacist’s education for optimal professional practice.

This topic was already addressed in 2009 in the United States, as presented in a study by Poirier et al. [20] which describes the need for education to improve cultural competence, which are described as essential for good practice. The course presented in this paper is divided into seven competencies such as the process of cultural competence in health service delivery, cultural awareness, or communication skills with families. This teaching is associated with an improvement of cultural competence, even if its organization and implementation remain substantially challenging.

The 2012 US study by Galal et al. [21] shows the importance of emotional competences for pharmacists, for good patient management. Such skills can be improved and should therefore be integrated into training curricula. For the authors, these skills are part of the process of student development which leads to become a “good” professional. In 2012, the American study by Okoro et al. [22] supports this need for student education: while minorities are more aware of cultural skills, all students need to be educated to understand health disparities. In 2013, Sales et al. [23] described the teaching methods to develop cultural competences and from 2014, studies were carried out to evaluate and improve them [24,25]. Similarly, in 2017, a study conducted by Jacob et al. [26] among students shows that religion and beliefs influence health and treatment. It also reveals that spirituality and religion influence professional practice.

#### 3.2.2. Professionalism

In 2010, a literature review [27] highlights the importance of communication skills for optimal patient management and the apprehension that it can generate in pharmacists. Practical teaching with patient simulation is an effective teaching strategy. In 2015, Mylrea et al. highlight the importance of professionalism training throughout pharmacist studies [28]. The authors define standards adapted to needs so that all students develop the same skills, taking into account the evolution of the profession.

Three articles address simultaneously cultural competences and professionalism. Cultural competences enable the development of professionalism. In 2013, a German study tested the link between communication skills among pharmacy students and better care of patients with dementia [29]. This study shows that considering a patient as a human being rather than as a patient can change the patient/pharmacist relationship and improve patient care. An American study in the same year emphasizes the importance of adapting communication to the public, especially to people with literacy difficulties [30]. They discuss the term “health literacy” and the need to use simple, non-technical language, which is sometimes a difficulty.

In 2016, an American article on this issue examines how to continue to integrate cultural skills into practice as medical technology evolves [31]. How can empathy be put into the digital communication that is developing more and more? Such cultural competencies must continue to exist with the development of new technologies and the evolution of the pharmacist profession.

Finally, in 2018, a Malaysian study questions healthcare professionals about ethics training for city professionals [32]. Following a series of workshops on ethics, a questionnaire was conducted to evaluate the benefit of ethics training. It is highlighted that the patient is obviously at the center of professionals’ interest but this is balanced with strong daily commercial pressure. Training on ethics is necessary. Such training can be considered in other countries.

Two American studies simultaneously address ethics and professionalism. The 2014 study (see (Appendix A)), which focuses on pharmacy students as well as professionals, highlights the importance of bioethics and professionalism in the pharmaceutical curriculum [33]. It is also mentioned that these two notions have been present in national recommendations since 2004. Finally, the 2015 study insists on the importance of introducing ethics and good pharmacy practices in the first year of the curriculum [34].

### 3.3. Interprofessional Collaborations and Integration of a Multidisciplinary Team

This last theme deals with the contribution of human and social sciences to inter-professional relations. Interprofessional collaboration is defined below; it is included in the curriculum for pharmacy students in order to confront them as early as possible with multidisciplinary work. This also acknowledges the role of pharmacists in public health strategies, a role which differs from one country to another.

Interprofessional Collaboration and Multidisciplinary Team Integration is described in 2018 by El-Awaisi et al. who recall what Interprofessional Education (IPE) is as given by the Centre for the Advancement of Interprofessional Education (CAIPE): it is defined as “two or more professions learning with and from each other to improve collaboration and quality of care”. They also echo World Health Organization’s definition of professional collaboration: “Collaborative practice in health care settings occurs when several health care providers from different professional backgrounds provide comprehensive services by working with patients, families, caregivers, and communities to provide the highest quality care in all settings” [35]. This collaboration is necessary in the face of the increasing complexity of health and illness. WHO requires that students be introduced to it during their training to avoid reluctance to collaborate in their future professional practice. This review of original literature specific to pharmacy shows that students perceive the need for interprofessional education in their curricula. However, this paper also describes certain limitations, such as the views of other professionals, particularly physicians.

As of 2013, IPE was practiced in the United States. Indeed, Goldstone et al. report a study in which pharmacy students integrate a multidisciplinary team in a psychiatric department [36]. This study allowed students to obtain more than 200 h of experience as a member of a multidisciplinary team and allowed the student to be trained on practical cases. The authors conclude that this should be implemented in other institutions. Another study in the same year highlighted the value of joint nurse/pharmacist training in diabetes management [37]. Students take the same courses which also allows professionals to improve their knowledge of other professions. The study found that the implementation of this type of course is feasible and effective, and that this course improves patient management and enhances interprofessional collaboration.

In 2014, a study in Tasmania, Australia, and New Zealand found that students perceive IPE as useful in their professional practice [38]. However, its teaching needs to be optimized, particularly by integrating it early in the curriculum with simple concepts. This study shows that IPE increases students’ knowledge of other professionals and their roles, and allows them to learn to interact with them earlier. In 2016, an American study looks at factors that may influence interprofessional collaboration [39]: cultural origins may modify interprofessional socialization with apprehensions that will hinder communication between professionals.

In Great Britain, the advantages of joint physician/pharmacist training in the case of polymedication in the elderly [40] is also studied. In a 2016 report, this training, which includes practical cases, is shown to provide optimal quality care and better global and personalized patient management. It enables students to learn to work with other professionals and to manage a team.

In 2016, a study in Arabic-speaking Middle Eastern countries evaluates IPE [41]. Inequalities exist between countries, particularly in relation to a lack of knowledge of the specificities of the different healthcare professions, particularly their role. It is difficult for a professional to explain to another professional the specific skills related to their specialty. It is noted by the authors that the main responding countries are Qatar, Jordan, Lebanon, and Saudi Arabia and that there is no response from Iraq and Yemen where political tensions are present. The geopolitics of the country therefore plays a huge role in health management and the development of health policy as shown in previous sections of the presented article. The paper from 2016 also describes the importance of support from faculty organizations to implement these courses, to enhance knowledge and to develop the necessary skills to adopt a positive attitude toward IPE and interprofessional collaborations. There is also a need to move beyond the professional culture that can be a source of interprofessional rivalries. Limiting factors such as time and money must also be considered in order to improve the implementation of IPE in the curriculum.

In 2017, the United States conducted a study on professional collaboration in dental care [42]. This study highlights the pharmacist’s coordinating role and its importance for the proper use of the medication and the reduction of adverse effects. It also describes the pharmacist’s involvement in the screening of certain diseases such as hypertension, diabetes, and so on. The authors conclude that pharmacists facilitate coordination and have a role in patient orientation. In this context, they recommend a review of the educational programs to better train future pharmacist to embrace these new roles.

## 4. Discussion

Our results show that the different organizations of health systems, combined with the diversity of university courses, contribute to a greater or lesser integration of human and social sciences in pharmaceutical practice in different countries. The status and role of pharmacists also play a role. However, a lack of knowledge of the professional role of pharmacists can make it difficult to interpret and ascertain the impact of the human and social sciences in certain countries. Nevertheless, the contribution of human and social sciences to pharmaceutical practice and education is key in terms of the strategic development of this profession within their new missions relating to public health and health promotion [43]. For example, pharmacists need to build knowledge and develop competences on how to behave towards and engage in a relationship with the patient, which implies for them to shift from a biomedical approach of health to a holistic and systemic approach to people’s health within their life ecosystems [44]. This requires to not restrain training to technical and specialized skills, but to integrate communication, counseling, and partnership skills [44,45]. Human and social sciences offer great and nuanced contributions to the field of health promotion. Our results support this assumption in the field of pharmacy in the sense that human and social sciences contribute to inform and develop inter-professional collaboration, which is one of the key competences pharmacists are required to develop in the current state of practice. The motivation of practitioners, or future practitioners, is essential in this process and it appears that students have grasped the importance of such integrated and interprofessional modes of practice. In this respect, interprofessional education will lead to optimal patient care.

Human and social sciences may also contribute to address and respond to new health reforms while encompassing and taking into account patients’ needs. More effective disease management implies that health determinants are considered [46,47]. Patients are seen as the human being at the center of care, which does impose certain degrees of shift in professional identity when it comes to healthcare professions, and which has to be integrated into curricula and pedagogical stances. At this point, the pressing issue may not be whether or not to include different types of competencies which relate to human and sciences, but rather what competences are key to pharmacy practices, as pointed out about other healthcare professions [48]. Most of the articles mentioned above address training or focus on students, and their perception of their role, and of their training. Human and social sciences should be integrated into the initial training of healthcare professionals in order to raise awareness of cultural differences, to learn to work in multidisciplinary teams or to adapt communication modes. Such integration during under- and postgraduate studies and in continuing education could contribute to counterbalance some of the resistances experienced later on in professional practices.

However, our results highlight that the integration of human and social sciences into training is very different between developed and developing countries, which have set different public health priorities. Indeed, in developing countries, the safety of the medicine circuit is a priority, as is the quality of professional training. In the United States and in Europe, pharmacists’ work is focused on the quality of professional practice. Disparities do exist; however, the United States is well ahead in introducing human and social sciences into university curricula, and into the evaluation of such curricula with a view to enhance the efficiency of the training, while European countries are still trying to integrate human and social sciences into initial training. The United States demonstrates great ethnic and cultural diversity, which therefore naturally puts the interest for human and social sciences in question, but also the difficulties which can emerge in this process. Among the potential barriers and difficulties, we argue that the difficulty to evaluate the benefits of such new paradigms is a key issue. It is necessary to evaluate this contribution in professional practice, which also requires the identification of clear learning outcomes [48]. This evaluation in could also take place to explore healthcare costs/gains, based on standardized and reliable criteria that have yet to be identified.

Another point is that advocacy from political decision-makers is fundamental. Human and social sciences should be taught systematically in all healthcare universities so that every professional receives the same education and patients can benefit from equal treatment. In order to set up new training courses, states decision-makers, and university organizations need to be involved in providing both human and financial resources. From an international perspective, it appears the state of progress in this area is very different between countries. The evolution of any professional practice is a slow process. Changes have to be considered on a long-term scale. Such changes imply raising awareness, changing mentalities of field professionals, introducing teacher training in early training, and ensuring that the economic means to implement such new curricula are provided. It naturally requires collaboration between health systems, health professionals, and teachers. As a final point, let us mention Svensson et al. (2012), who warned against the potential consequences of a change to the training of pharmacists, pointing to a risk of such changes being counterproductive. A key question is thus pending and would have to be addressed in future research: will this shift produce innovators and leaders or resistors to change and followers? [49]

## 5. Conclusions

If clinical pharmacy (drug reconciliation, pharmaceutical interviews, and therapeutic education) becomes a discipline that can be integrated into the human and social sciences, it responds to a need expressed by patients but also by society in a difficult economic context. The practice of pharmacy is undergoing major changes, even if there is great variability between countries as to what the profession of pharmacist is and how it is evolving. Nevertheless, it requires pharmacists to receive broader training in ethics and health education, for a personalized approach based on the patient rather than on the medicine. Cooperation between health professionals is essential here. The human and social sciences contribute to the development of its pharmaceutical activities; they require the development of new skills which must be explained and taught from the initial course.

## Appendix A. Study Characteristics

**Article**	**Year of Publication**	**Type of Publication**	**Country**	**Sub-Theme**
*Main topic: Strategic evolution of the pharmacist profession*				
Hassali MA, et al. Social pharmacy as a field of study: the needs and challenges in global pharmacy education. [10]	2011	Commentary	Malaysia	Global disparities in public health
Law MG, et al. Addressing the global need for public health clinical pharmacists through student pharmacist education: a focus on developing nations. [12]	2017	Commentary	USA	Global disparities in public health
Palaian S, et al. Initiation of social pharmacy research in Nepal: our experiences. [11]	2011	Commentary	Nepal	Global disparities in public health
Bush AA, et al. Identifying Shared Values for School-Affiliated Student Organizations. [18]	2017	Empirical study	USA	Adapting training to change
King AE, et al. A Required Online Course with a Public Health Focus for Third Professional Year Pharmacy Students. [16]	2015	Empirical study	USA	Adapting training to change
Offiong CY, et al. The role of colleges and schools of pharmacy in the advent of Healthy People 2020. [9]	2010	Commentary	USA	Adapting training to change
Teagarden JR. Well connected: pharmacy education and the humanities. [17]	2013	Commentary	USA	Adapting training to change
Youmans S, et al. Clinical pharmacy to meet the health needs of Tanzanians: education reform through partnership across continents (2008–2011). [13]	2012	Commentary	Tanzania	Adapting training to change
American College of Clinical Pharmacy, et al. Cultural competency in health care and its implications for pharmacy part 3A: emphasis on pharmacy education, curriculums, and future directions. [15]	2013	Commentary	USA	Global disparities and adapting training to change
Nunes-da-Cunha I, et al. A Comparison of Patient-Centered Care in Pharmacy Curricula in the United States and Europe. [14]	2016	Empirical study	Europe/USA	Global disparities and adapting training to change
David PM, et al. [The evolution of pharmaceutical practice within the context of changing health territories: How can social sciences contribute to pharmaceutical education?]. [8]	2010	Commentary	France	Pharmacist and public health
Momas I. [Public health and the pharmacist: a challenge of importance regarding training]. [7]	2009	Commentary	France	Pharmacist and public health
*Main topic: Relationship skills towards the patient*				
Saw PS, et al. A practical approach toward teaching ethics to community pharmacists. [32]	2018	Empirical study	Malaysia	Ethic
Cooper LA, et al. Pharmacy students’ perceptions of cultural competence encounters during practice experiences. [24]	2014	Empirical study	USA	Cultural competences
Echeverri M, et al. Racial Dynamics and Cultural Competence Training in Medical and Pharmacy Education. [25]	2017	Empirical study	USA	Cultural competences
Galal S, et al. Development and assessment of social and emotional competence through simulated patient consultations. [21]	2012	Empirical study	USA	Cultural competences
Godwin JN Jr. An enhanced appreciation of cultural competency: Applying knowledge at home and abroad. [19]	2017	Commentary	USA	Cultural competences
Jacob B, et al. First-year Student Pharmacists’ Spirituality and Perceptions Regarding the Role of Spirituality in Pharmacy Education. [26]	2017	Empirical study	USA	Cultural competences
Okoro ON, et al. Clinical cultural competency and knowledge of health disparities among pharmacy students. [22]	2012	Empirical study	USA	Cultural competences
Poirier TI, et al. A cultural competency course for pharmacy students. [20]	2009	Empirical study	USA	Cultural competences
Sales I, et al. A comparison of educational interventions to enhance cultural competency in pharmacy students. [23]	2013	Empirical study	USA	Cultural competences
Chen AM, et al. Impact of a health literacy assignment on student pharmacist learning. [30]	2013	Empirical study	USA	Cultural competences and professionalism
Terry C, et al. The Emerging Issue of Digital Empathy. [31]	2016	Commentary	USA	Cultural competences and professionalism
Zimmermann M. Integrating medical humanities into a pharmaceutical care seminar on dementia. [29]	2013	Empirical study	Germany	Cultural competences and professionalism
Horton ER, et al. A Novel Structured Format for Engaging Pharmacy Students in Bioethics Discussions. [33]	2014	Empirical study	USA	Ethic and professionalism
Smith MG, et al. Early Introduction to Professional and Ethical Dilemmas in a Pharmaceutical Care Laboratory Course. [34]	2015	Empirical study	USA	Ethic and professionalism
Mesquita AR, et al. Developing communication skills in pharmacy: a systematic review of the use of simulated patient methods. [27]	2010	Review article	Brazil	Professionalism
Mylrea MF, et al. Professionalization in Pharmacy Education as a Matter of Identity. [28]	2015	Commentary	Australia	Professionalism
*Main topic: Interprofessional collaborations and integration of a multidisciplinary team*				
Branch-Mays GL, et al. An Interprofessional Education and Collaborative Practice Model for Dentistry and Pharmacy. [42]	2017	Empirical study	USA	In professional practice
El-Awaisi A, et al. A comprehensive systematic review of pharmacy perspectives on interprofessional education and collaborative practice. [35]	2018	Review article	Qatar	In professional practice
LaRochelle JM, et al. Racial Differences in Communication Apprehension and Interprofessional Socialization in Fourth-Year Doctor of Pharmacy Students. [39]	2016	Empirical study	USA	In professional practice
El-Awaisi A, et al. Interprofessional education in the Arabic-speaking Middle East: Perspectives of pharmacy academics. [41]	2016	Empirical study	Arabic-speaking Middle East	Disparities
Anderson E, et al. Interprofessional learning on polypharmacy. [40]	2016	Empirical study	Royaume-Uni	Right for the training
Gilligan C, et al. Recommendations from recent graduates in medicine, nursing and pharmacy on improving interprofessional education in university programs: a qualitative study. [38]	2014	Empirical study	Australia TasmaniaNew Zealand	Right for the training
Goldstone LW, et al. An interprofessional psychiatric advanced pharmacy practice experience. [36]	2013	Empirical study	USA	Right for the training
Pittenger AL, et al. An interprofessional diabetes experience to improve pharmacy and nursing students’ competency in collaborative practice. [37]	2013	Empirical study	USA	Right for the training
